# Peripheral tissue oximetry: comparing three commercial near-infrared spectroscopy oximeters on the forearm

**DOI:** 10.1007/s10877-013-9507-9

**Published:** 2013-08-30

**Authors:** Simon Hyttel-Sorensen, Trine Witzner Hessel, Gorm Greisen

**Affiliations:** Department of Neonatology, Rigshospitalet, University Hospital Copenhagen, Blegdamsvej 9, 2100 Copenhagen, Denmark

**Keywords:** Near infrared spectroscopy (NIRS), Oxygenation, Muscle, Medical device

## Abstract

Estimation of regional tissue oxygenation (rStO_2_) by near infrared spectroscopy enables non-invasive end-organ oxygen balance monitoring and could be a valuable tool in intensive care. However, the diverse absolute values and dynamics of different devices, and overall poor repeatability of measurements are a problem. The aim of the present study is to test the hypothesis that INVOS 5100C, FORE-SIGHT and NONIN EQUANOX 7600 have similar properties concerning absolute values, repeatability, and sensitivity to changes in rStO_2_. To test repeatability the sensors were repositioned 20 times during hemodynamic steady state on the adult forearm. Afterwards six vascular occlusions by inflation of an upper arm cuff were done to achieve low oxygenation in the forearm. Absolute values were compared by repeated-measures ANOVA, repeatability was estimated by the within-subject standard deviation, S_w_, and response to changing oxygenation by the down slope of rStO_2_ during vascular occlusion in the respective arm. 10 healthy adults, 21–29 years old, with double skinfolds on the forearm less than 10 mm participated. The median rStO_2_ was 70.7 % (interquartile range (IQR) 7.7 %), 68.4 % (IQR 8.4 %), and 64.6 % (IQR 4.8) with INVOS, NONIN, and FORE-SIGHT, respectively, the median rate of decline was 13.2 %/min (IQR 9.6), 22.8 %/min (IQR 18.0), and 10.8 %/min (IQR 6.0), and the same-site repeatability was 2.9 % (95 % CI 2.4–3.3), 4.6 % (CI 3.9–5.3), and 2.0 % (CI 1.7–2.3). INVOS gave significantly higher steady state values than FORE-SIGHT, and NONIN had the steepest decline in rStO_2_, but the poorest repeatability. Two measures of signal-to-noise were similar among devices. This suggests that good repeatability comes at the expense of low sensitivity to changes in oxygenation. Values of rStO_2_ on the forearm from INVOS, NONIN and FORE-SIGTH cannot be used interchangeably.

## Introduction

Near infrared spectroscopy (NIRS) enables estimation of the regional tissue haemoglobin oxygen saturation (rStO_2_). rStO_2_ is a weighted average of the saturation in arterial and venous blood in the illuminated tissue. The arterial-to-venous ratio is about 25:75 and rStO_2_ is therefore closer correlated to the venous than to the arterial saturation [1]. Thus NIRS is a non-invasive, continuous estimate of the regional oxygen delivery-consumption balance and could potentially detect low regional blood flow that would otherwise go undetected [2]. However implementation into standard clinical care has yet to be seen. This is likely due to lack of convincing outcome data and randomized trials in combination with rather costly equipment. Furthermore previous studies have shown that a repeated measurement can vary up to 14 percentage points (95 % CI) [3–5]. This large intra-subject variability is an issue that needs improvement, before the potential of the technology can be fully realized.

Near infrared spectroscopy oximetry is based on the fact that hemoglobin exist in two main forms, oxyhemoglobin (O2Hb) and deoxyhemoglobin (HHb), each having its own particular optical absorption characteristics. Most commercial devices apply continuous wave spectroscopy and use a multi-distance approach measuring the reflected light attenuation at several wavelengths as a function of distance from the light source. Assumptions regarding the wavelength dependence of scattering is necessary to derive the spectral shape of absorption coefficient and thereby deduce rStO_2_ [6]. These assumptions in combination with assumptions about the water content and the different light emitter and detector geometry probably account for the differences between devices.

It is well established that peripheral muscle rStO_2_ is a hemodynamic variable, that at least on group level differs between the healthy and the severely ill [7, 8], and is related to clinical relevant outcomes [7, 9, 10]. While these studies were done with different NIRS oximeters on different sites on the body the issue of generalizability is a hindrance to widespread clinical implementation. A recent review by Scheeren et al. [11] addresses some of the current strengths and limitations of the technology.

The new generation of NIRS sensors are FDA approved to claim reliable absolute values meaning that clinical validation studies have shown sufficient precision compared to a weighted average of arterial and jugular bulb saturation [12]. This should translate into better repeatability of measurements and suggests a significant step forward for the technology. Previous studies comparing different NIRS devices focus primarily on steady state comparison of mean values, whereas no estimates are given for repeatability or sensitivity to changing oxygenation. When comparing different devices all three parameters are of importance: the absolute rStO_2_ values during the normal state, the changes in rStO_2_ when oxygenation is altered, and the repeatability, i.e. the similarity of repeated measurements.

The aim of the present study is test the hypothesis that three commercial NIRS devices for peripheral measurements have similar absolute values of rStO_2_. Secondary outcomes include repeatability of measurements and slopes of changes in rStO_2_ during a vascular occlusion test. The devices compared are INVOS 5100c™ (Covidien, Boulder, CO, USA), a trend monitor, FORE-SIGHT™ (CAS Medical Systems Inc, Branford, CT, USA), and NONIN EQUANOX 7600™ (Nonin Medical Inc, Plymouth, MN, USA) that both are FDA approved for absolute measurements.

## Materials and methods

This study was approved by the local Ethics Committee (Journal no. H-3-2012-004) and conducted at the national university hospital, Rigshospitalet, Denmark. Written informed consent was obtained from the participants before inclusion. The study was registered at clinicaltrials.gov (ID NCT01552785).

Recruitment of healthy volunteers was accomplished by an ad in a local university paper. Inclusion criteria included normal health and a skinfold thickness of less than 10 mm on the lower arm measured by the Harpeden caliper to minimise the contribution of subcutaneous tissue. Exclusion criteria were pregnancy, hypertension, peripheral vascular disease, and local skin disease.

All measurements were done with subjects placed in upright position with the lower arm at heart level. The outline of each sensor was marked by a pen on the upper part of the flexor muscles of the lower arm to ensure that the sensors were applied to the same spot each time.

### Absolute values and repeatability

**Fig. 1:**
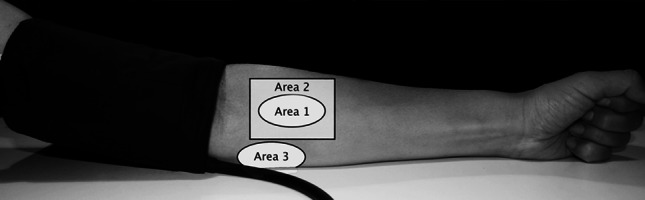
The areas on the arm used for the measurements. *Area 1* is the marked area for same site repeated measuresments, *Area 2* is the area of repeated measurement at different sites, and *Area 3* is the marked area of sensor position during cuff occlusion

The absolute rStO_2_ values of each device and repeatability of measurements during hemodynamic steady state were tested with one device at a time. First 20 seconds (s) of measurement was repeated 10 times at the marked site with total sensor lift up between repeats (Area 1 Fig. 1). As sensor repositioning in the clinical setting is likely to be at a slightly different site the sequence was repeated with the sensor being re-positioned at slightly differing sites around the area (Area 2 Fig. 1). This way an estimate of both the ‘optimal’ and the ‘clinical’ repeatability is achieved. This gave 2 × 10 steady state measurements with each device on each participant.

### Sensitivity to changes in oxygenation

**Fig. 2:**
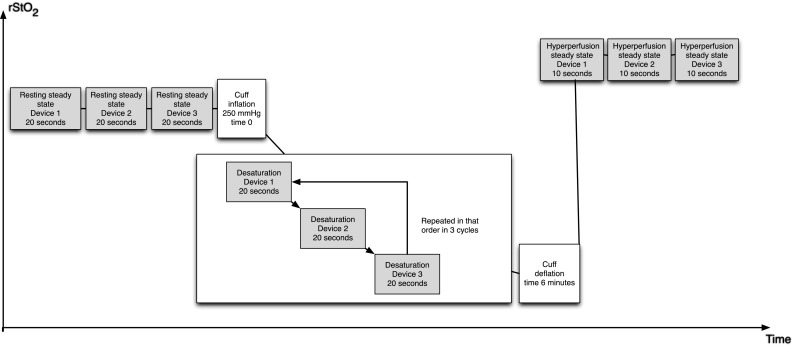
Schematic presentation of the measurements during cuff inflation and deflation

Secondly testing of device sensitivity to changing oxygenation was done with a vascular occlusion test inflating a cuff around the upper arm at heart level to more than 250 mmHg. 20 s of baseline rStO_2_ with each device was recorded before the cuff was inflated. 10 s after cuff inflation the different sensors were applied at the same site (Area 3 Fig. 1) sequentially to obtain three 1-min cycles of measurement of down-sloping rStO_2_. Each device measured 20 s within each cycle. After cuff deflation each device recorded 10 s of rStO_2_ during the short steady state of subsequent hyperperfusion (Fig. 2). The sequence of the three devices was changed between occlusions and six occlusions per subject were done ensuring a balanced design.

As the adhesive strength of the sensors wears off with repeated measurements the sensors were held in place by hand. This was found to be the most reliable way to get stable measurements, while great care was taken not to induce pressure on the tissue while avoiding possible source-to-detector light piping. All sensors were positioned so that the light path was parallel to the longitudinal direction of the muscles below. The experiment typically lasted 180 minutes in total per subject.

### NIRS instruments

The three NIRS devices tested all use the multi-distance approach.

INVOS 5100c™ with adult SomaSensor™ (SAFB-SM) uses two LED sources (730 and 810 nm) at one position and two photodiode detectors at a distance of 3 and 4 cm, and “subtracts” the short distance signal from the longer distance in order to diminish the contribution of the skin and scalp.

NONIN EQUANOX™ 7600 with EQUANOX Advance™ Sensor, Model 8004CA™ uses four LEDs (730, 760, 810, and 880 nm) in each of two positions 6 cm apart with two photodiode detectors 2 cm apart in between in a symmetrical design.

FORE-SIGHT™ Cerebral Oximeter with the medium sensor uses a 4-wavelength (690, 779, 808, and 850 nm) laser source delivered to the sensor through an optical fibre and two photodiode detectors 1.25 and 4 cm from the source, respectively.

We will refer to the measured regional tissue oxygenation as rStO_2_ irrespective of instrument.

### Statistical analysis

The a priori hypothesis was that the instruments have similar absolute values.

Sample size was calculated to have a power of 80 % to detect a 5-percentage points difference between absolute values with a standard deviation of 5 % and a paired design. A repeated measures general linear model tested differences in absolute values between devices with device as within-subject factor. Residual plots were visually assessed to assure reasonable fit. If data were either highly skewed or the variance was heterogeneous among groups a related samples Friedman test was used. If a significant device effect was found, post hoc pair-wise comparison was carried out.

Repeatability for each instrument was determined by one-way ANOVA with subject as the factor. The within-subject standard deviation, S_w_, was then estimated from the square root of the residual mean square. The 95 % CI being ±1.96 times S_w_/root(2n(m − 1)), where n is number of subjects and m the number of observations per subject.

To assess the dynamic properties of each device a simple, least square linear regression was applied to each 20 s sample of rStO_2_ recording during occlusion based on the approximation that the decline in rStO_2_ over time is linear during the first 3 min of desaturation [13, 14].

Two different approaches to estimate a signal-to-noise ratio (SNR) for each device were used. For each device on each subject a SNR was derived from the mean of the first cycle slopes divided by the same-site within-subject standard deviation (slope-SNR). Secondly data from the steady-state measurements were used. From a one-way ANOVA with the 20 s measurement as factor the spontaneous within-measurement fluctuations in rStO_2_ was used as signal while the between measurements variance was the noise term (variance-SNR). All data were log-transformed before the SNR analyses to achieve normal distribution.

Data were tested for normality with the Sharpiro Wilk test. Results from data with normal distribution are reported as mean with 95 % CI, whereas all other results are given as median with interquartile range. A *p* value below 0.05 was considered significant. Bonferroni adjustment was applied where appropriate.

All data was analysed in a Matlab versus R2011b (The MathWorks, Inc, Natick, MA, USA) script. All statistical tests were done in SPPS statistics version 20 (IBM, Corp, Armonk, NY, USA).

## Results

10 subjects (5 male/5 female) healthy adults with an age range of 21–29 years and a median skinfold of 5.2 mm (IQR 3 mm) were included.

### Absolute values

**Fig. 3:**
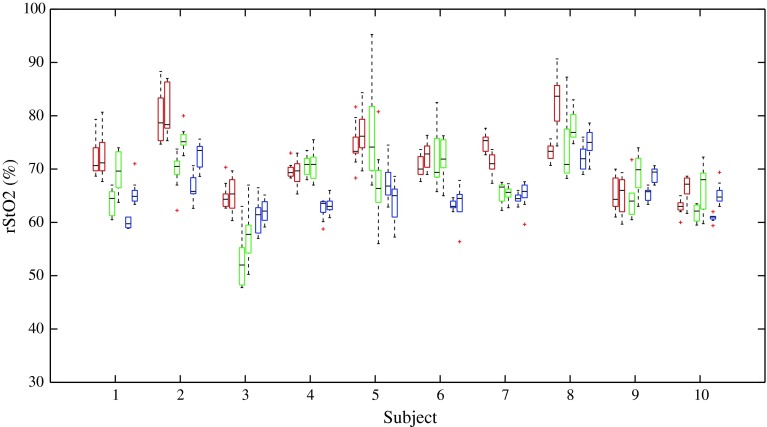
The results from the steady-state same-site and various-site measurements. INVOS is *red*, NONIN *green*, and FORE-SIGHT *blue* (presented in that order from left to right). Each device-subject pair has the same-site measurements to the left and various-site measurements to the right. The *boxes* represents the interquartile ranges, the *whisker* the range, and the *horizontal bar* the median. Outliers are marked by a *red plus symbol*

During resting steady state the overall median rStO_2_ was 70.7 % (IQR 7.7 %), 68.4 % (IQR 8.4 %), and 64.6 % (IQR 4.8) with the INVOS, NONIN, and FORE-SIGHT, respectively (Table 1; Fig. 3). In pairwise comparison only INVOS versus FORE-SIGHT were significantly different (*p* = 0.01).

**Table 1:** The absolute values of rStO_2_ of each device

Device	Position	N	Median (%)	IQR (%)
INVOS	Same site	100	70.5	8.0
	Various sites	100	71.0	8.5
	Total	200	70.7	7.7
NONIN	Same site	100	67.0	8.3
	Various sites	100	69.5	8.6
	Total	200	68.4	8.4
FORE-SIGHT	Same site	100	63.8	4.7
	Various sites	100	65.8	6.3
	Total	200	64.6	4.8
Total	Same site	300	66.7	8.5
	Various sites	300	68.6	8.5
	Total	600	67.7	8.8

During post-cuff-deflation reperfusion the median rStO_2_ was 85.3 % (IQR 11.3 %) with INVOS, 88.0 % (IQR 14.0 %) with NONIN, and 77.7 % (IQR 10.5 %) with FORE-SIGHT. The FORE-SIGHT was significantly lower than the two other devices (*p* < 0.0001) in both pair-wise comparisons.

### Repeatability

The repeatability of FORE-SIGHT was best, while the repeatability of NONIN was worst (Table 2; Fig. 3). There was no difference between same-site repeatability and various-site repeatability.

**Table 2:** The repeatability of same site and various site repositionings

	Same site		Various sites	
	S_w_ (%)^a^	CI (%)^b^	S_w_ (%)^a^	CI (%)^b^
INVOS	2.9	2.4–3.3	3.6	3.0–4.1
NONIN	4.6	3.9–5.3	3.8	3.3–4.4
FORE-SIGHT	2.0	1.7–2.3	2.4	2.0–2.7

^a^S_w_: the within-subject standard deviation of the 10 repeated measurements

^b^CI: 95 % confidence interval

### Sensitivity to changes in oxygenation

**Fig. 4:**
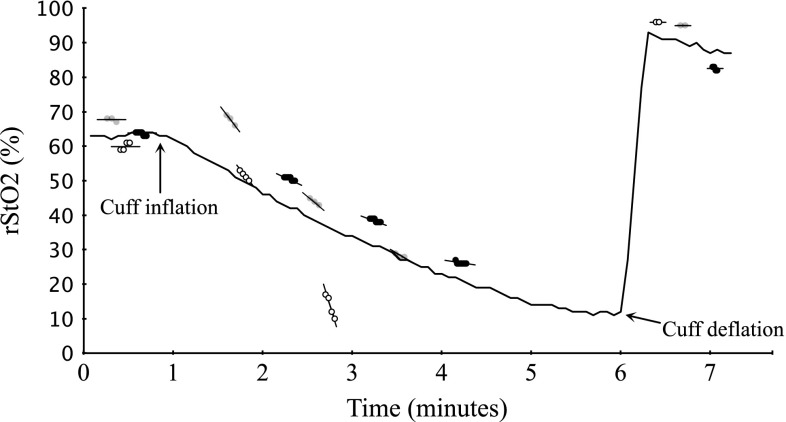
An example of the first vascular occlusion test on a subject. The *grey dots* are INVOS; the *black dots* are FORE-SIGHT; the *blank dots* are NONIN. The continuous tracing is from another occlusion, but is included to illustrate the typical oxygenation trend. The *horizontal lines* represent steady state measurements. The *regression lines* are included for each of the 20 s measurements during the occlusion. Note that NONIN only have two measurements during the occlusion due to loss of signal

The slopes of desaturation were steepest with NONIN (*p* < 0.0001) in both pair-wise comparisons, while the slopes of INVOS and FORE-SIGHT were similar (Table 3). Often both NONIN and INVOS reached their lower limit of recording before the start of third cycle of measurement giving an unequal number of observations per device, thus both analysis of data from all three cycles and only the first cycle was done giving the same main results (Fig. 4).

**Table 3:** The dynamic ranges

	First cycle slopes (%/min)	IQR (%/min)	All slopes (%/min)	IQR (%/min)
INVOS	13.2	9.6	10.4	8.5
NONIN	22.8	18.0	21.8	18.0
FORE-SIGHT	10.8	6.0	8.5	5.7

### Signal-to-noise ratio

Both the cuff-induced slope-SNRs and spontaneous variance-SNRs were similar among devices. Slope-SNRs were 22.0 min^−1^ (CI 18.8–25.7), 23.8 min^−1^ (CI 19.7–28.8), and 22.0 min^−1^ (CI 17.8–27.1) with INVOS, NONIN, and FORE-SIGHT, respectively (*p* = 0.5). The variance-SNRs were 0.26 (CI 0.19–0.36), 0.24 (CI 0.18–0.32), and 0.21 (CI 0.16–0.26) with INVOS, NONIN, and FORE-SIGHT, respectively (*p* = 0.3).

## Discussion

### Important findings

The median resting steady state absolute values of the three oximeters were within 6 percentage points, whereas the values during deoxygenation were markedly different. The repeatability of FORE-SIGHT was best, while NONIN had the steepest slope of change during deoxygenation. The three devices had similar signal-to-noise ratios. Figure 3 illustrates this as the NONIN can be seen to have both the largest within-subject as well as between-subject variance.

### Appraisal of method

This study was designed to compare three commercial NIRS oximeters and hypothesized that the devices give similar readings. The three sensors have quite different light source and detector geometries and it could be argued that the volumes of tissue being examined are so different, that systematic difference are to be expected. However, if the overall goal is to provide a single, simple measure of tissue oxygenation for clinical use then similarity among oximeters is necessary.

The strength of our method is that it both gives an estimate of repeatability together with a measurement of the sensitivity to changing oxygenation. The method is easily reproducible and allows fast comparison of several instruments in healthy subjects with almost no risk. Vascular occlusion by cuff is a simple and robust way of inducing maximal tissue deoxygenation, allowing efficient estimation of device sensitivity. The response to cuff inflation and subsequent deflation probably varied during the six runs in each subject, but as our test design with each devices measuring on every round in a balanced manner, it is unlikely to have caused bias.

One important limitation of our study is that total occlusion of blood flow by a manually inflated cuff is not physiological. It results in a slight increase in blood volume due to the arterial inflow while the cuff pressure builds up and the venous outflow is already occluded. Thus the arterial to venous volume ratio (A:V ratio) is likely to decrease. In systemic hypoperfusion and sepsis the A:V ratio probably increases so the translation to clinical measurements may not be straightforward. Moreover the adhesive strength of the sensors wears off with repeated repositionings so the sensors were held by hand. Small differences in local pressure squeezing blood from superficial vessels cannot be completely excluded, but great care was taken and it is unlikely to have influenced the measurements much. Lastly it should be noted that these sensors are primarily intended for continuous monitoring whereas spot measurements are the cornerstone of this study. There are, however, no physiological or technical reason to believe that a reliable baseline measurement cannot be achieve within 10 s of stable readings as the used NIRS devices do not require calibration procedures or warm up.

### Comparison with previous studies

Our results compare reasonably well with previous findings. The median baseline values measured in the range of 65–75 % were slightly higher with the INVOS. This is in accordance with other studies [15–17]^.^ While in our previous similar study INVOS 5100C with the same adult sensor had a similar mean rStO_2_ of 70.2 ± 6.7 % [5]^.^

It is interesting that the S_w_s of all instruments were quite low, meaning better repeatability than expected. Most studies have reported repeatability of about 5–6 % [3, 4, 18, 19]. In respect to the INVOS we have no good explanation for the difference in repeatability compared to our own, previous study, since both methods and subjects were similar.

To our knowledge these instruments haven’t been tested and compared on muscle before. Data from the brain suggest that the FORE-SIGHT has better repeatability than INVOS [16]. Comparing the three instruments with a reference rStO_2_ calculated from jugular bulb and arterial blood in the human adult the accuracy root mean square was 4.26 % with FORE-SIGHT, 9.69 % with INVOS, and 6.27 % with NONIN [15]. While the accuracy root mean square is not straightforwardly comparable to the S_w_ in the present study the differences between instruments should be the same. It is thus again noticeable that INVOS performed better in our study.

The InSpectra Model 325 (Hutchinson Technology Inc., Hutchinson, MN, USA) is a NIRS device specifically designed for peripheral measurements only. Comparing the slopes of desaturations in %/min of INVOS and FORE-SIGHT from present study with a study using the InSpectra on the forearm and thenar muscle shows reasonable agreement [20].

∆rStO_2_ in a recent study comparing the changes in rStO_2_ during a circumferential pneumatic head cuff inflation to estimate the contribution of extra-cranial scalp tissue were 16.6 ± 9.6, 11.8 ± 5.3, and 6.8 ± 6.0 % with INVOS, FORE-SIGHT, and NONIN, respectively [17]. Interestingly the ratio of ∆rStO_2(INVOS)_/∆rStO_2(FORE-SIGHT)_ are similar in our study. This suggests that the differences in dynamics found on the arm could be similar on the head, and that the differences in so-called removal of extra-cranial tissue contamination as presented in the study by Davie and Grocott could be simply a matter of different sensitivity to overall oxygenation changes in the tissue. The results with the NONIN are not comparable to present study as the classic Model 8000CA sensor was used, that previously has been found to be less sensitive than the new Model 8004CA [15], which was the sensor used in our study.

### Clinical relevance

It is a key problem of NIRS tissue oximetry that there is no reference standard. We did not draw arterial and venous blood samples, as it is uncertain how well the venous saturation reflects the tissue oxygenation in a state of no flow. The fact that NONIN and INVOS often reached their lower boundary values while FORE-SIGHT showed continuing deoxygenation at a level of 20–30 % suggests that NONIN and INVOS overestimate true deoxygenation in the adult human forearm. It is not certain that FORE-SIGHT is closer to the ‘true’ values, but at least the continued decline in rStO_2_ is likely to represent truly changing oxygenation.

It is noticeable that the results suggest that the variability of repeated measurements is independent of whether the sensors are repositioned at same site or at a different site. The implies at least on the lower arm that the exact sensor position is less important and that the source of variance is not so much differences in muscle oxygenation between adjacent tissues, but perhaps more a combination of physiologic fluctuations and optical heterogeneity on a smaller scale—such as hair and subcutaneous blood vessels in the light path.

When NIRS oximetry is used for trend monitoring the repeatability of measurement is less important, but good repeatability is paramount if NIRS is to be used as a spot measurement or if the monitoring is started when the patient status is uncertain, e.g. on admission to critical care. Moreover in critical care the sensor has to be repositioned during prolonged monitoring to avoid skin damage. The dynamic sensitivity in relation to repeatability is equally important since it is an estimate of the reliability of potential hypoxic levels of tissue oxygenation. If, for the sake of argument, it is assumed that a jugular bulb saturation below 50 % indicates cerebral ischemia, then with a A:V ratio of 25:75 this theoretically corresponds to rStO_2_ 61 %, if the SaO_2_ is 95 %, thus with a S_w_ of 5 % a spot measurement should be below (61 % − 1.96 × 5 %) 52 % to be more than 95 % certain that the tissue is actually ischemic and above (61 % + 1.96 × 5 %) 71 % to be certain that it is not. If in addition the sensitivity to changes is low the risk of undetected true hypoxia will be high. The actual changes in oxygenation induced by cuff inflation-deflation, is unknown in the current setup, but it is safe to say that the three tested devices do not agree about the magnitude of changes. A pragmatic approach would be to care less about the closeness to the ‘true’ values, but to settle for the device with the best combination of repeatability and sensitivity to changing oxygenation. However our results suggest that high sensitivity comes at the expense of good repeatability, as our estimates of signal-to-noise ratios for each device were similar.

The lack of a reference standard is a drawback for the technology and it would be a large step forward if an international standard could be determined. One problem with in vivo testing as done in the present study is that results may or may not be generalizable to other tissues. It is thus important to test and compare the commercial available NIRS equipment on different sites in different populations. In-vitro phantom testing could also prove useful, but has not been widely used with commercial equipment, even though it could perhaps provide a possible framework for a future standardization procedure.

## Conclusion

In conclusion we found that INVOS 5100C with the adult SomaSensor, NONIN EQUANOX 7600 with sensor 8004CA, and FORE-SIGHT with medium sensor all had pair-wise differences in either median absolute values, repeatability, and/or dynamic measurements. A standardized testing procedure would be a step forward for the technology.
